# Identification of the biological functions and chemo-therapeutic responses of ITGB superfamily in ovarian cancer

**DOI:** 10.1007/s12672-024-01047-4

**Published:** 2024-05-30

**Authors:** Jiawen Han, Lin Lyu

**Affiliations:** Department of Nutrition, Jinshan Hospital, Fudan University, 1508 Longhang Road, Jinshan District, Shanghai, 201508 China

**Keywords:** ITGB, Biological function, Ovarian cancer, Chemo-therapeutic, Responses, Prognostic prediction

## Abstract

**Background:**

Patients with ovarian cancer (OC) tend to face a poor prognosis due to a lack of typical symptoms and a high rate of recurrence and chemo-resistance. Therefore, identifying representative and reliable biomarkers for early diagnosis and prediction of chemo-therapeutic responses is vital for improving the prognosis of OC.

**Methods:**

Expression levels, IHC staining, and subcellular distribution of eight ITGBs were analyzed using The Cancer Genome Atlas (TCGA)-Ovarian Serous Cystadenocarcinoma (OV) database, GEO DataSets, and the HPA website. PrognoScan and Univariate Cox were used for prognostic analysis. TIDE database, TIMER database, and GSCA database were used to analyze the correlation between immune functions and ITGBs. Consensus clustering analysis was performed to subtype OC patients in the TCGA database. LASSO regression was used to construct the predictive model. The Cytoscape software was used for identifying hub genes. The ‘pRRophetic’ R package was applied to predict chemo-therapeutic responses of ITGBs.

**Results:**

ITGBs were upregulated in OC tissues except ITGB1 and ITGB3. High expression of ITGBs correlated with an unfavorable prognosis of OC except ITGB2. In OC, there was a strong correlation between immune responses and ITGB2, 6, and 7. In addition, the expression matrix of eight ITGBs divided the TCGA-OV database into two subgroups. Subgroup A showed upregulation of eight ITGBs. The predictive model distinguishes OC patients from favorable prognosis to poor prognosis. Chemo-therapeutic responses showed that ITGBs were able to predict responses of common chemo-therapeutic drugs for patients with OC.

**Conclusions:**

This article provides evidence for predicting prognosis, immuno-, and chemo-therapeutic responses of ITGBs in OC and reveals related biological functions of ITGBs in OC.

**Supplementary Information:**

The online version contains supplementary material available at 10.1007/s12672-024-01047-4.

## Introduction

Ovarian cancer (OC) is the second lethal malignancy among female reproductive cancers [1]. According to the National Center for Health Statistics, the estimated number of new cases of OC is 19,710, and the number of deaths of OC is 13,270 [2]. Owing to the lack of typical symptoms at an early stage, most (75%) patients are diagnosed at an advanced stage [1]. The standard management for OC is surgery combined with platinum and paclitaxel. However, up to 70% of patients at stage III-IV face chemoresistance and recurrence in 3 years [3]. Despite developments in the treatment of OC, the overall survival (OS) of OC did not show improvement and the 5-year OS rate was less than 50% [4, 5]. Therefore, the investigation of biomarkers for early diagnosis and overcoming chemoresistance are vital for improving the prognosis of patients with OC. Immune therapy showed limitations in OC for years. Recently, a phase II clinical study made a breakthrough, which for the first time showed the benefits of immune checkpoint inhibitors (ICIs) in treating advanced OC. This study remarkably inspired more studies about immunotherapies of OC [6].

Integrin β superfamily (ITGBs) includes eight members, Integrin Subunit Beta (ITGB) 1 (ITGB1), ITGB2, ITGB3, ITGB4, ITGB5, ITGB6, ITGB7, and ITGB8. Currently, ITGBs were reported to predict prognosis in hepatocellular, pancreatic, and non-small cell lung cancer [7]. ITGA and ITGB superfamily members have been reported to predict prognosis in high-grade serous ovarian cancer [8, 9], but the biological functions, oncologic characteristics, and therapeutic responses need to be revealed in OC.

This study revealed for the first time the alteration of eight ITGBs in OC and metastatic sites of OC. Additionally, we analyzed the biological functions, tumor-infiltrating immune cells, immuno-, and chemo-therapeutic responses of ITGBs in OC using bioinformatic analyses combined with experiments.

## Materials and methods

### Cell culture

Human epithelial OC cell lines OVCAR-3 (RRID: CVCL_0465) were obtained from American Type Culture Collection (ATCC, Manassas, VA, USA) and were cultured in Roswell Park Memorial Institute‐1640 (PRIM-1640) (Corning Inc., New York, USA), supplemented with 20% fetal bovine serum (FBS, Invitrogen, Carlsbad, CA, USA). Nontumorous human immortalized ovarian surface epithelial cell line IOSE-80 (RRID: CVCL_5546) was purchased from FuHeng BioLogy (FuHeng BioLogy, Shanghai, China) and cultured in PRIM-1640 supplemented with 10% FBS. All cell lines were authenticated by short tandem repeat (STR) analysis and were routinely detected to be pathogen-free and mycoplasma-negative.

### Database collection and data analysis

The workflow of the study is presented in Supplementary Fig. 1 (Fig. S1). Gene expression matrix and clinical information of OC patients were downloaded from The Cancer Genome Atlas (TCGA) -Ovarian Serous Cystadenocarcinoma (OV) (https://www.cancer.gov/tcgaA total of 379 OC samples in TCGA-OV were used for the consequent analyses. Healthy samples of The Genotype-Tissue Expression (GTEx) were downloaded from the UCSC Xena website (https://xenabrowser.net/datapages/). Among 9783 samples of GTEx, only 88 samples were healthy ovary tissue. Therefore, the gene-expression matrix of 88 normal ovary tissues and 379 OC samples were used for gene difference analysis. The clinical information of 379 OC samples was used to analyze the subgroup classification, prognostic correlation, and predictive model construction of ITGBs. GSE26712, GSE133859, and GSE131978 were downloaded from the National Center for Biotechnology Information (NCBI, https://www.ncbi.nlm.nih.gov/gds/) Gene Expression Omnibus (GEO) DataSets for differential expression analysis [10–12]. Detailed information about the GEO DataSets is provided in Table S1. R version 4.1.1 (2021-08-10) was used for statistical computing and graphics in this paper.

RNA-seq data of GSE131978 was from GPL570 and GPL96. Therefore, the batch effect is removed first using ‘limma’ packages (limma_3.50.3) [13]. After the batch removal process, the merged GSE131978 contains 39 samples of OC. The 39 samples include 9 omental metastatic samples with short-term survival, 18 ovarian cancer tissues with short-term survival, and 12 ovarian cancer tissues with long-term survival. The 12 ovarian cancer tissues with long-term survival were excluded for comparability.

### Quantitative real‐time polymerase chain reaction (qRT-PCR) and RNA extraction

Total RNA was extracted using an RNA-Quick Purification Kit (Yishan Biotechnology Co., Ltd, Shanghai, China) in OVCAR-3 and IOSE-80 cell lines. Complementary DNA (cDNA) was synthesized using a first-strand complementary DNA synthesis kit (Roche). cDNA was then amplified using the 7300 Real-Time PCR System (Applied Biosystems, Thermo Fisher Scientific, Inc., MA, USA) with BeyoFast TM SYBR Green qPCR Mix (2X, High ROX Beyotime). Actin was used as an internal control. The primer sequences are listed in Table S2.

### Kaplan–Meier survival analysis

The PrognoScan website was used for Kaplan–Meier (K-M) survival analysis of ITGBs in OC, based on the DUKE-OC database (http://dna00.bio.kyutech.ac.jp/PrognoScan/) [14]. The K-M survival analysis between high- and low-score groups was performed using the "survminer" (survminer_0.4.9) R package [15].

### Consensus clustering, principal component analysis (PCA), and construction of a predictive model

Consensus clustering was applied to identify distinct cancer subgroups based on the expression matrix of ITGBs using the k-means clustering algorithm. K-means is a type of partition clustering method that needs a specific value of k. Each k has its corresponding consensus matrix and number of clusters. The optimal consensus matrix can distinguish the cancer clearly and obviously. Therefore, the optimal matrix corresponding k is the optimal one. The number of clusters, and their stability, were determined by the consensus clustering algorithm using the "ConsensuClusterPlus" R package (ConsensuClusterPlus_1.36.0) [16] based on the TCGA-OV database. We performed 1,000 times repetitions to guarantee the stability of our classification.

Least Absolute Shrinkage and Selection Operator (LASSO) regression is commonly used to construct the prognostic model for patients with cancer [17]. For constructing the predictive model, the ITGBs-based consensus cluster analysis divided the TCGA-OV database into two subgroups. A total of 114 differential genes was obtained between the two subgroups. To further determine the correlation between the 114 genes and the prognosis of OC patients, they were used to construct the predictive model using the ‘glmnet’ R package (glmnet_4.1–7) [18]. The TCGA-OV database was divided into train and test groups randomly. The train group was used to construct the predictive model while the test group was used to testify the efficiency of the model. The value of lambda (λ) facilitates the construction of the predictive model. The λ value with the minimum mean-squared error is the optimal predictive model. In the process of constructing the predictive model, univariate regression analysis was conducted first to identify 13 genes associated with the overall survival rate of OC patients (Table S3), which were considered potential predictive factors. Subsequently, LASSO regression and multivariate regression analysis were performed based on the above 13 genes to identify 4 independent predictive genes and their coefficients (Table S4). The coefficient of each gene was used to calculate the predictive score of each sample, which could predict the prognosis of OC patients. K-M survival and ROC analyses in the test group were performed to verify the predictive model's efficiency.

### Nomogram

Clinical information (age, stage, and grade) of the TCGA-OV database and predictive scores of the predictive model were used to construct a nomogram to predict the OS rate of patients with OC using the "rms" R package (rms_6.7–0) [19]. The calibration curve confirmed the coherence between the observed and predicted OS rate, which is used to evaluate the accuracy and performance of the predictive model.

### GO, KEGG, and GSVA enrichment and genomic mutation status

TCGA-OV database was divided into high- and low-ITGB1 groups according to the median value of ITGB1 in the TCGA-OV database. Then different-expressed genes between the two groups were produced using the ‘limma’ [13] R package (FC > 1, P-value < 0.05). These genes were called co-expression genes of ITGB1. In the same way, we got co-expression genes of the rest seven ITGBs. For Gene ontology (GO) and Kyoto Encyclopedia of Genes and Genomes (KEGG) analyses, a total of 268 co-expression genes of eight ITGBs were used for the enrichment using ‘org.Hs.eg.db’ (org.Hs.eg.db_3.14.0), ‘enrichplot’ (enrichplot_1.14.2), and ‘clusterProfiler’ (clusterProfiler_4.2.2) R packages [20–22]. Then the circle graphs of GO and KEGG enrichments were plotted by the ‘GOplot’ (GOplot_1.0.2) R package [23]. The genomic mutational status of ITGBs was downloaded from the cBioPortal website (https://www.cbioportal.org/). As to the GO and KEGG analyses of 15 hub genes, the 15 genes were uploaded onto the Database for Annotation, Visualization and Integrated Discovery (DAVID) website (https://david.ncifcrf.gov) for the enrichment [24] and ‘ggplot2’ (ggplot2_3.5.1) was used for plotting the graph [25].

Gene Set Variation Analysis (GSVA) was performed using the ‘limma’ (limma_3.50.3), ‘GSEABase’ (GSEABase_1.56.0), and ‘GSVA’ (GSVA_1.42.0) packages [13, 26, 27] and plotted using the ‘pheatmap’ (pheatmap_1.0.12) package [28]. Co-expression genes of ITGB1, ITGB3, and ITGB8 which were acquired as mentioned above were used for GSVA.

### Hub gene analysis

Protein–protein interaction (PPI) network data was analyzed and downloaded from the STRING website (https://string-db.org/) based on a total of 268 co-expression genes of eight ITGBs [29]. Then the interaction data was imported into Cytoscape software (Version 3.8.2, RRID: SCR_003032) and processed to draw the PPI network graph and calculate the top 15 hub genes using the degree parameter in the cytoHubba plugin.

### Correlations between immune responses and eight ITGBs

The TIDE scoring file of the TCGA-OV database was downloaded from the Tumor Immune Dysfunction and Exclusion (TIDE) database (http://tide.dfci.harvard.edu/) [30]. The immune-scoring data between high and low expression ITGBs groups were analyzed using ‘limma’ (limma_3.50.3) and ‘ggpubr’ (ggpubr_0.6.0) R packages [13, 31].

The correlations between the immune stimulator, MHC, immune inhibitor, and eight ITGBs were downloaded from the Gene Set Cancer Analysis (GSCA) database (https://guolab.wchscu.cn/GSCA/#/) [32].

### Prediction of chemo-therapeutic responses

First, samples from the TCGA-OV database were divided into high- and low-ITGB expression groups, according to the median of each ITGB. The ‘pRRophetic’ package (pRRophetic_0.5) [33] was downloaded from GitHub (https://github.com/) and used to calculate the association between the IC_50_ of common chemo-therapeutic drugs of OC and expression levels of each ITGB.

### HPA database

The human protein atlas (HPA) website (https://www.proteinatlas.org/) [34, 35] was used to analyze the expression levels of ITGBs in ovarian cancer tissue and normal ovarian tissue which were presented as immunohistochemistry staining images. Moreover, the website provided subcellular distribution and three-dimensional (3D) structure prediction of ITGBs as well. Immunofluorescence images of ITGBs in U2OS, U-125MG, HaCat, and Rh30 cell lines were all from the HPA database.

### Statistical analysis

GraphPad Prism 8.0 (RRID: SCR_002798, GraphPad Software Inc.) was used to analyze all data and the data was presented as the mean $\pm$ SD. The student's *t*-test was used for a two-group comparison. The Spearman rank test was used to analyze the correlations between two factors. The Cox hazard regression model was applied to calculate the hazard ratio (HR). The Kaplan–Meier survival curve analysis was performed using the Log-rank test. P-value < 0.05 was considered as statistically significant.

## Results

### Characteristics of ITGBs family in OC

**Fig. 1:**
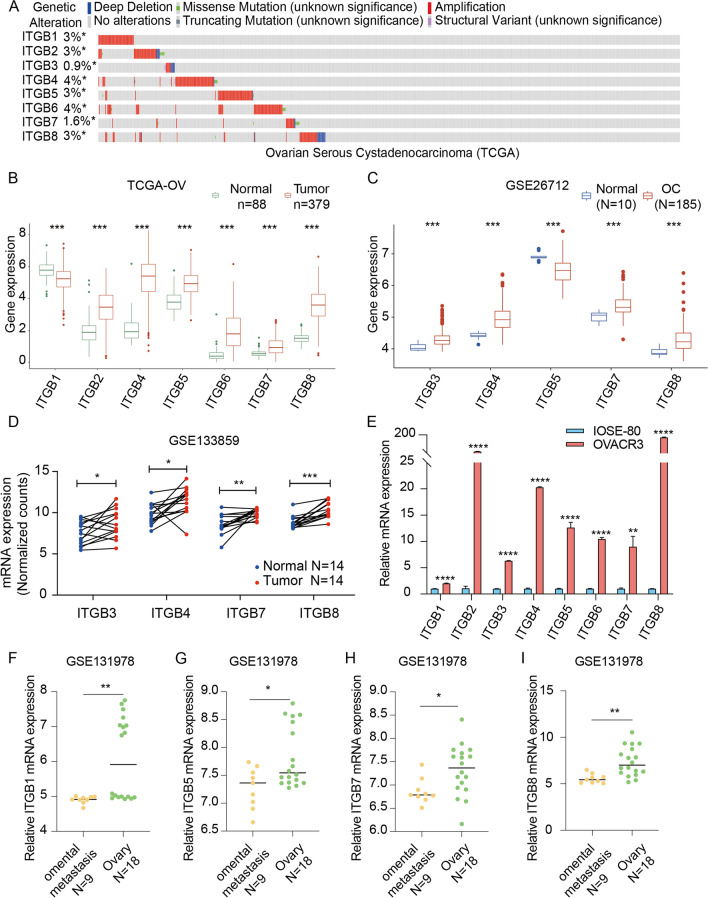
Genomic mutant status and expression patterns of ITGBs in OC. **A** Genomic mutant status of ITGBs from the cBioPortal website. Amplification is the most evident genetic alteration. **B** Expression levels of ITGBs in 88 normal ovarian tissues and 379 OC tissues from TCGA-OV and GTEx databases. **C** Expression levels of ITGBs in the GSE26712 DataSet. **D** Paired expression levels of ITGBs in the GSE133859 DataSet. **E** qRT-PCR experiment of mRNA expression levels of ITGBs in IOSE-80 and OVCRA-3 cells. n = 3. Data was presented as mean $\pm$ SD. Unpaired student's *t*-test was used for the comparison. **F**–**I** Expression levels of ITGB1, ITGB5, ITGB7, and ITGB8 in GSE131978 DataSet. P < 0.05 was considered as significant. *, P-value < 0.05; **, P-value < 0.01; ***, P-value < 0.001; ****, P-value < 0.0001

Amplification is the most common genetic alteration occurring in the eight ITGB superfamily members (ITGBs) in ovarian serous cystadenocarcinoma according to the TGCA-OV database, which explains elevated expression levels of ITGBs in OC to some extent (Fig. 1A). Data from TGCA-OV and GTEx showed that six ITGBs were significantly upregulated except ITGB1 and ITGB3 in OC (Fig. 1B). Data from the GSE26712 DataSet showed that ITGB3, ITGB4, ITGB7, and ITGB8 were upregulated and ITGB5 was downregulated in OC (Fig. 1C). GSE133859 provided paired OC samples, which demonstrated that ITGB3, ITGB4, ITGB7, and ITGB8 increased in ovarian tumor tissue compared with normal tissue (Fig. 1D). Results of qRT-PCR experiment showed that all eight members were upregulated in the ovarian cancer cell line (OVCAR-3) compared with the normal epithelial ovarian cell line (Immortal Ovarian Surface Epithelial Cell Line, IOSE-80) (Fig. 1E). However, the fold changes of ITGB1 and ITGB3 in OC cell line are less than 10, indicating a weak upregulation in OC. Data of omental metastatic tissue and tumor tissue in OC were obtained from GSE131978. The RNA-seq technology of GSE131978 was performed using two platforms as described in Table.S1. Therefore, the batch effect was removed (Fig.S2A, B) before different-expressed genes analysis. The result showed that ITGB1, 5, 7, and 8 decreased in omental metastasis tissue compared with OC tissue of patients with OC (Fig. 1F-I). Generally, ITGBs increased in TCGA-OV samples except ITGB1, consequent GEO DataSets showed that only ITGB3, 4, 5, 7, and 8 increased in OC samples compared with normal ovarian tissues. qRT-PCR experiment between the OC cell line and normal ovarian epithelial cell line proved all eight ITGBs increased in the OC cell line. Additionally, ITGB1, 5, 7, and 8 decreased in metastatic sites compared with primary tumor sites.

**Fig. 2:**
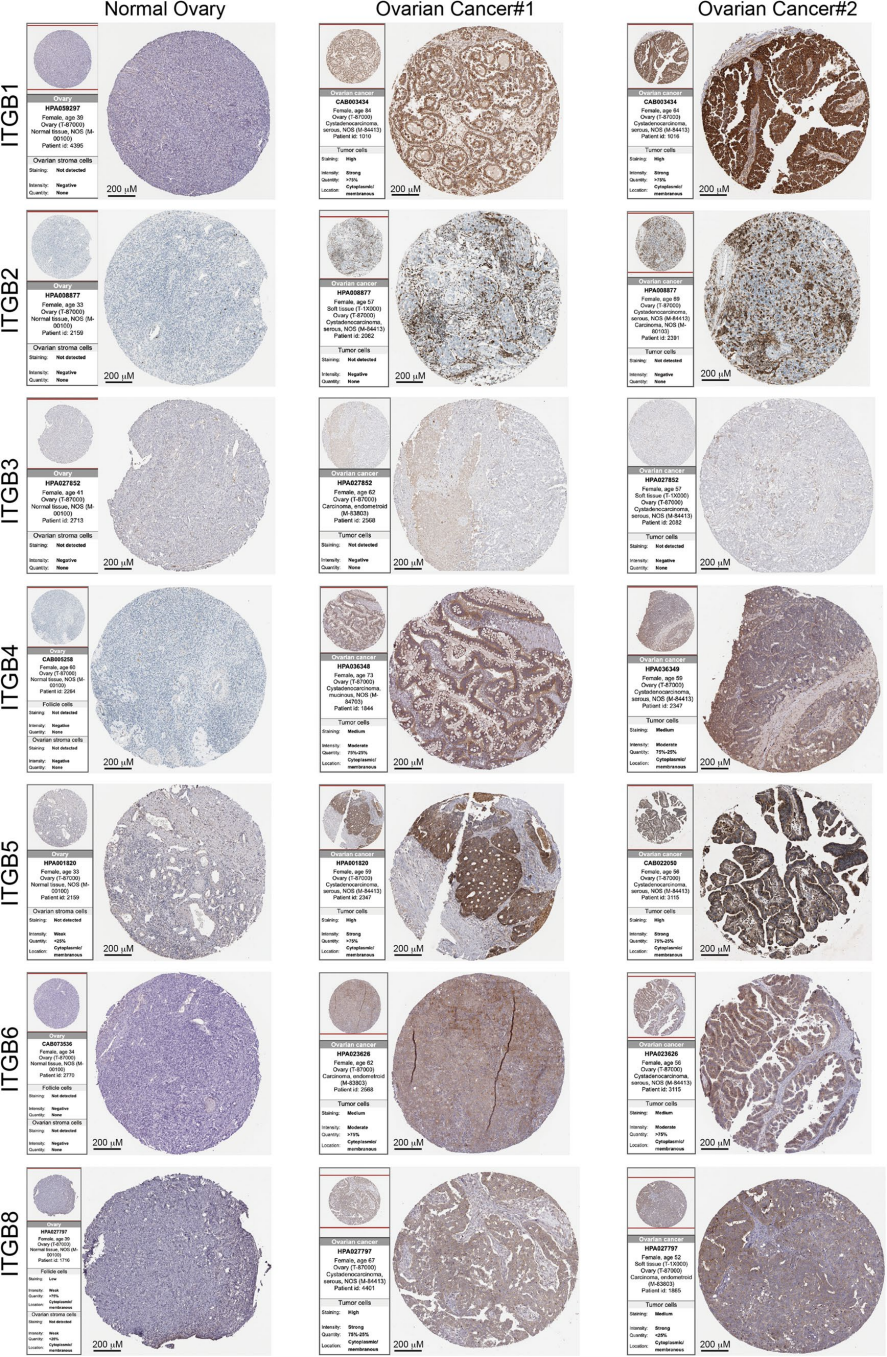
IHC staining images of ITGBs in OC from the HPA database. IHC staining images of seven ITGBs were downloaded from the HPA database in normal ovarian tissue and OC tissue. Origins of antibodies and tissue were presented in the left annotation beside each IHC image. Ovarian cancer#1 and Ovarian cancer#2 represent different OC samples. Scale bar, 200 µM

**Fig. 3:**
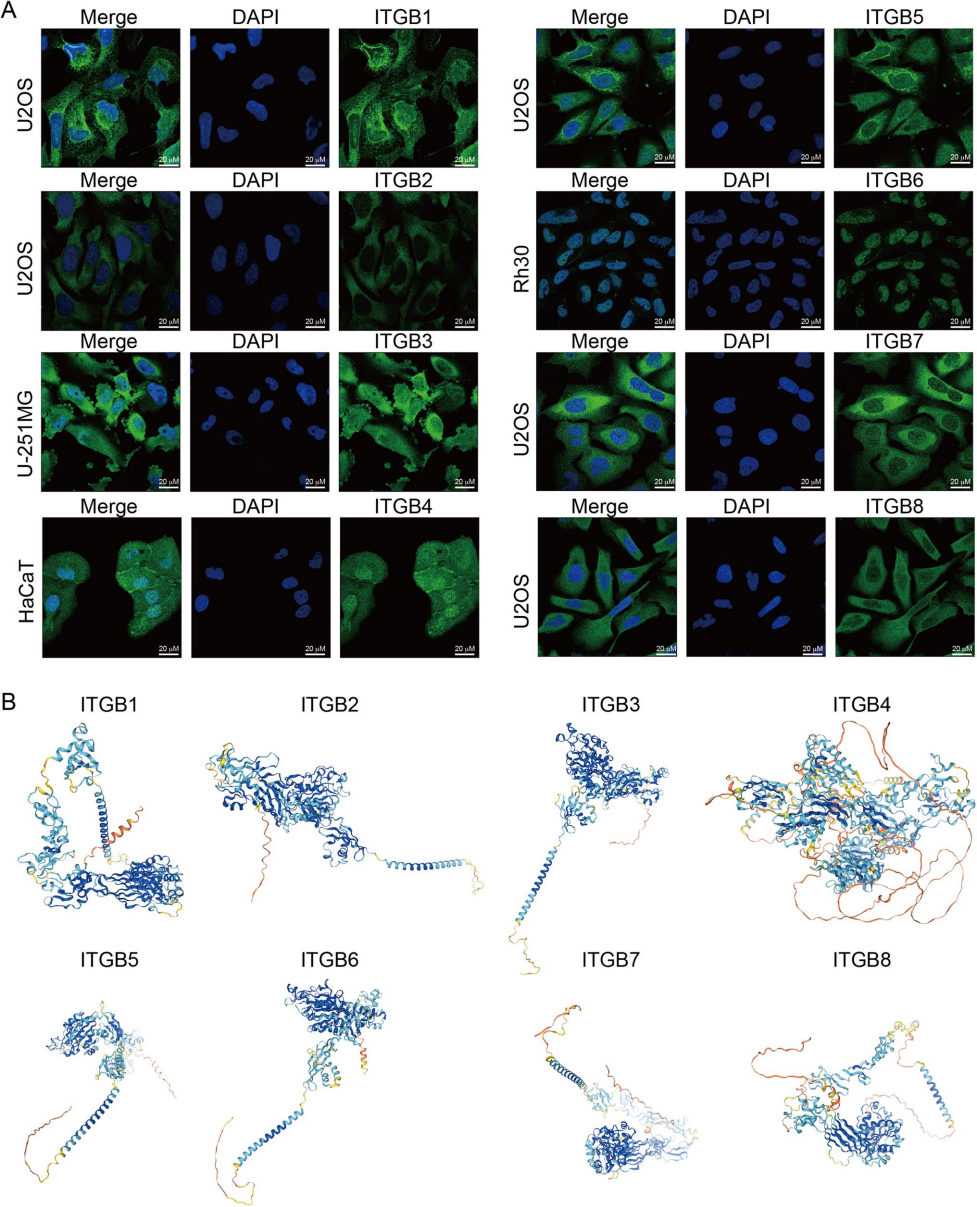
Subcellular distribution and 3D structure of eight ITGBs. **A** Immunofluorescence images of eight ITGBs in cell lines were downloaded from the HPA database. Scale bar, 20 µM. U-2 OS, sarcoma cell line. U-251MG, glioblastoma cell line. HaCaT, human immortalized epidermal cell line. Rh30, rhabdomyosarcoma cell line. The antibody for ITGB1 is CAB003434. The antibody for ITGB2 is HPA016894. The antibody for ITGB3 is HPA027852. The antibody for ITGB4 is HPA036348. The antibody for ITGB5 is CAB0220505. The antibody for ITGB6 is HPA023626. The antibody for ITGB7 is HPA042277. The antibody for ITGB8 is HPA027796. **B** The 3D structure of eight ITGBs was downloaded from AlphaFold, version 2

The human protein atlas (HPA) provides IHC staining of proteins in normal tissue and tumor tissues. Concerning the results of HPA, IHC staining of ITGB1, 2, 3, 4, 5, 6, and 8 were acquired among normal ovarian tissues and OC tissues. IHC staining images from HPA showed that the above seven ITGBs had higher staining in OC tissues than in normal ovarian tissues (Fig. 2). The staining and intensity of the seven ITGBs were provided by the PHA database and were summarized in Table 1. According to the table, the staining and intensity of ITGB1, 2, 3, 4, 5, 6, and 8 were not detected and negative in normal ovarian tissues. The intensity of ITGB1, 5, and 8 are strong in OC tissues. However, the mRNA expression level of ITGB1 decreased in OC compared with normal ovary. Therefore, a potential post-translational modification mechanism might explain the inconsistency between the mRNA and protein expression levels of ITGB1 in OC. The intensity of ITGB4 and ITGB6 are moderate in OC tissues. Although IHC images of ITGB2 and ITGB3 showed positive staining, the annotations showed negative intensity in OC. Therefore, the protein level of ITGB2 and ITGB3 might be unchanged in OC. The left annotation of each IHC staining image exhibited the origin of the antibody and tissue. Additionally, the subcellular distribution of each ITGB was provided by HPA as well (Fig. 3A). U-2 OS is a sarcoma cell line in which immunofluorescence (IF) staining of ITGB1 is mainly distributed in the cytoplasm, especially in the perinuclear area. While IF images of ITGB2 showed even distribution in the cytoplasm in the U-2 OS cell line. U-251MG is a glioblastoma cell line in which ITGB3 is distributed both in the nucleus and cytoplasm, mainly in the cytoplasm. HaCaT is a human immortalized epidermal cell line in which ITGB4 is distributed both in the nucleus and cytoplasm, slightly more in the nucleus. ITGB5 exhibited a similar distribution as ITGB1 in the U-2 OS cell line. Rh30 is a rhabdomyosarcoma cell line in which ITGB6 is distributed mainly in the nucleus. Similarly, ITGB7 had an identical distribution as ITGB1 in the U-2 OS cell line. At the same time, ITGB8 is distributed mainly in the cytoplasm in the U-2 OS cell line. Generally, ITGB1, 5, and 7 had a similar distribution pattern, indicating potential similar functions of ITGB1, 5, and 7. While ITGB2, 3, and 8 had an identical distribution pattern. ITGB4 and ITGB6 are mainly distributed in the nucleus, which demonstrated a different biological function from the rest of ITGBs.

**Table 1:** IHC staining and intensity of ITGBs from the HPA database

ITGBs	Staining			Intensity		
	Normal ovary	Ovarian Cancer#1	Ovarian Cancer#2	Normal ovary	Ovarian Cancer#1	Ovarian Cancer#2
ITGB1	Not detected	High	High	Negative	Strong	Strong
ITGB2	Not detected	Not detected	Not detected	Negative	Negative	Negative
ITGB3	Not detected	Not detected	Not detected	Negative	Negative	Negative
ITGB4	Not detected	Medium	Medium	Negative	Moderate	Moderate
ITGB5	Not detected	High	High	Weak	Strong	Strong
ITGB6	Not detected	Medium	Medium	Negative	Moderate	Moderate
ITGB8	Not detected	High	Medium	Weak	Strong	Strong

The staining and intensity of each ITGB are provided by the HPA database. Ovarian cancer#1, Corresponding images in Fig. 2. Ovarian cancer#2, Corresponding images in Fig. 2

Additionally, HPA provided the 3D structure prediction of ITGBs from AlphaFold project version 2 (Fig. 3B). Proteins had specific structures for specific biological functions. ITGB1, 2, 3, 5, 6, and 7 had a similar 3D structure with a single-ended stretch-out α helix.

**Fig. 4:**
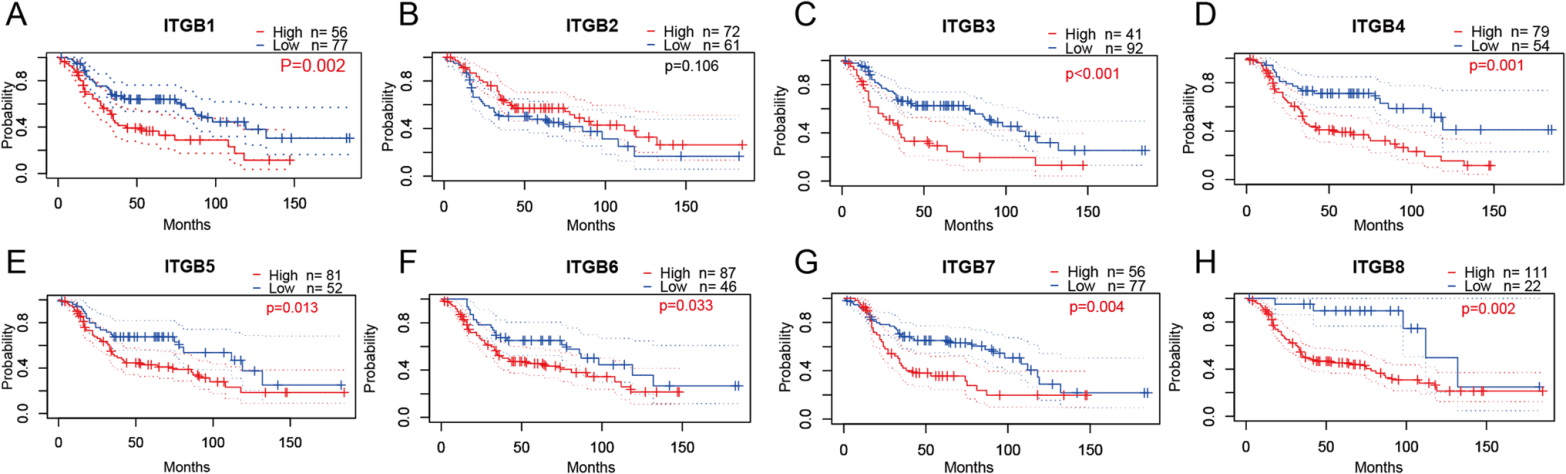
Kaplan–Meier survival analyses of ITGBs in OC. **A**–**H** K-M plots of ITGBs in OC were downloaded from the PrognoScan website. Low expression of ITGB1, 3, 4, 5, 6, 7, and 8 correlated with a favorable prognosis of patients with OC. P-value < 0.05 was considered as significant

Based on the DUKE-OC database, K-M survival analyses showed that low expression of ITGBs was favorable for the prognosis of patients with OC (Fig. 4A–H) except ITGB2. Univariate Cox analysis showed that ITGB1, 3, and 8 are risk factors for OC with a hazard ratio of 1.86 (1.14–3.04), 1.57 (1.07–2.30), and 1.18 (1.01–1.37) respectively (Table 2), indicating the solid role of ITGB1, 3, and 8 in predicting the prognosis of patients with OC.

**Table 2:** Univariate Cox analysis of ITGBs for overall prognosis (OS) in OC based on the TCGA-OV database

Gene	HR	HR.95L	HR.95H	P-value
ITGB1	1.86	1.14	3.04	0.013
ITGB2	0.93	0.81	1.08	0.352
ITGB3	1.57	1.07	2.3	0.022
ITGB4	1.96	0.68	5.66	0.215
ITGB5	1.1	0.77	1.57	0.587
ITGB6	1.06	0.79	1.42	0.706
ITGB7	1.34	0.97	1.85	0.079
ITGB8	1.18	1.01	1.37	0.037

P value < 0.05 was considered as significant

### Biological function enrichment of ITGB1, ITGB3, and ITGB8

**Fig. 5:**
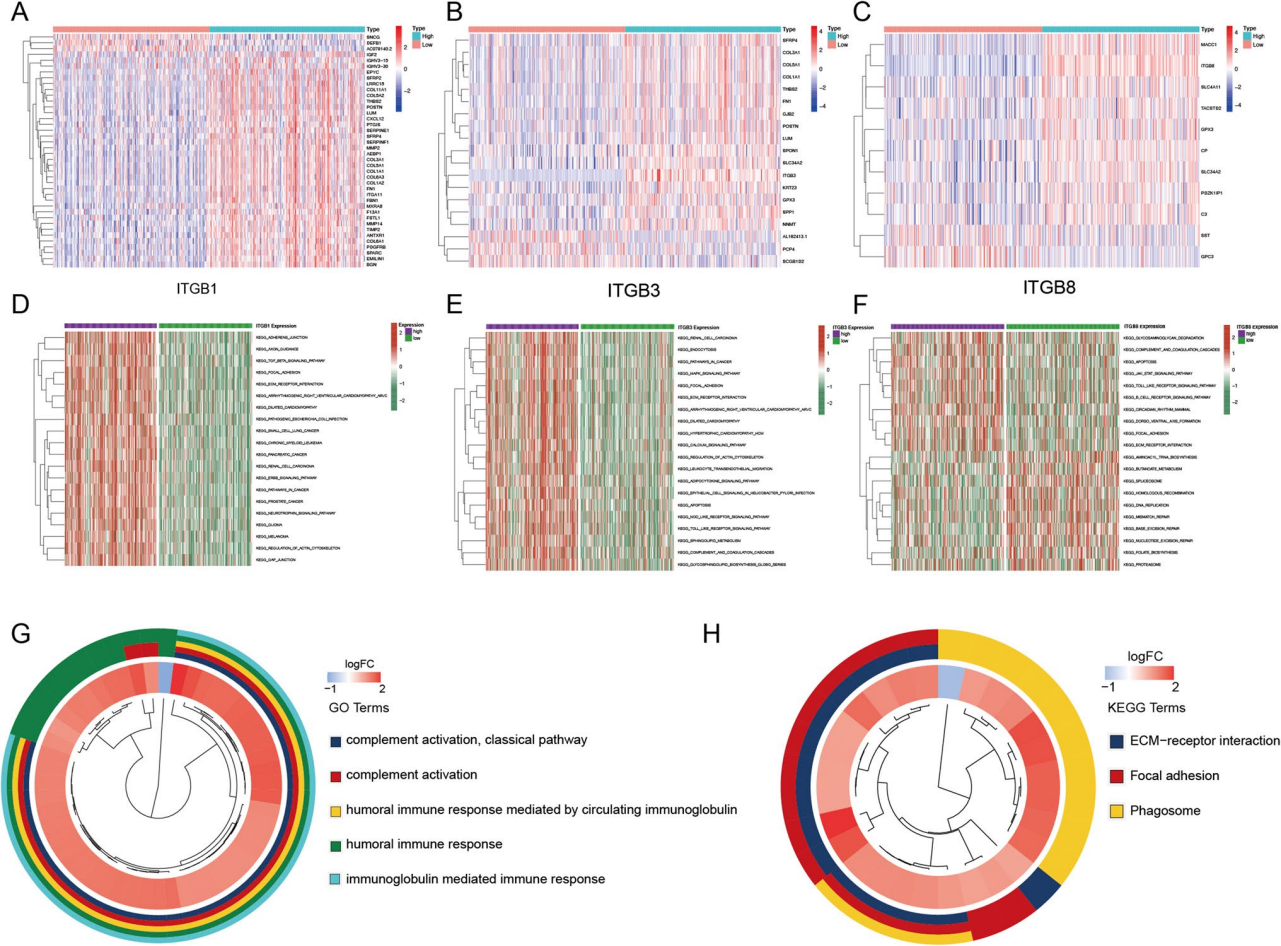
Biological function enrichment of ITGB1, ITGB3, and ITGB8. **A** Co-expression genes of ITGB1 were presented in the heatmap (FC > 1, P-value < 0.05). **B** Co-expression genes of ITGB3 were presented in the heatmap (FC > 1, P-value < 0.05). **C** Co-expression genes of ITGB8 were presented in the heatmap (FC > 1, P-value < 0.05). **D**–**F** GSVA was based on the above co-expression genes of ITGB1, ITGB3, and ITGB8. The ‘limma’, ‘GSEABase’, and “GSVA’ packages were involved in GSVA. (G, H) A total of 268 co-expression genes of eight ITGBs were used for the cluster analyses of GO and KEGG enrichment. The inner circle represents the input 268 genes. Blue indicates genes that were downregulated in high-ITGBs groups and negatively correlated with ITGBs, while red indicates genes that were upregulated in high-ITGBs groups and positively correlated with ITGBS. The outer ring consists of different colors representing GO terms or KEGG pathways as the annotation presented. Genes of the inner ring correspond to the colored outer ring, indicating their involvement in these GO terms or KEGG pathways. The cluster analyses of GO and KEGG indicate the corresponding correlations between genes and terms. The ‘‘org.Hs.eg.db’, ‘enrichplot’, and ‘clusterProfiler’ R packages were used for the analyses. The ‘GOplot’ package was used for the plot. GO, Gene Ontology. KEGG, Kyoto Encyclopedia of Genes and Genomes

According to Univariate Cox analysis, ITGB1, 3, and 8 are risk factors for OC. Therefore, it is interesting to figure out the potential biological functions of ITGB1, 3, and 8 in OC. First, the TCGA-OV database was divided into high- and low-ITGB1, high- and low-ITGB3, and high- and low-ITGB8 according to the median of ITGB1, 3, and 8 respectively. Then different-expressed genes between high and low groups of ITGB1, 3, and 8 were analyzed and presented as heatmaps respectively (Fig. 5A–C) (FC > 1, P-value < 0.05). Consequently, Gene Set Variation Analysis (GSVA) analysis showed that ITGB1 correlated with the TGF-β signaling pathway, extracellular matrix (ECM) receptor interaction, pathway in cancer, and regulation of actin cytoskeleton (Fig. 5D); ITGB3 correlated with endocytosis, focal adhesion, ECM receptor interaction, regulation of actin cytoskeleton, and apoptosis (Fig. 5E); while ITGB8 correlated with apoptosis, Janus kinase–signal transducer of activation (JAK-STAT) signaling pathway, ECM receptor interaction, focal adhesion, and mismatch repair (Fig. 5F). For GO and KEGG analysis, all co-expression genes of eight ITGBs were used. It’s demonstrated that humoral immune response, complement activation, ECM-receptor interaction, and focal adhesion were correlated with ITGBs (Fig. 5G, H).

### ITGBs correlated immune functions and immune responses

**Fig. 6:**
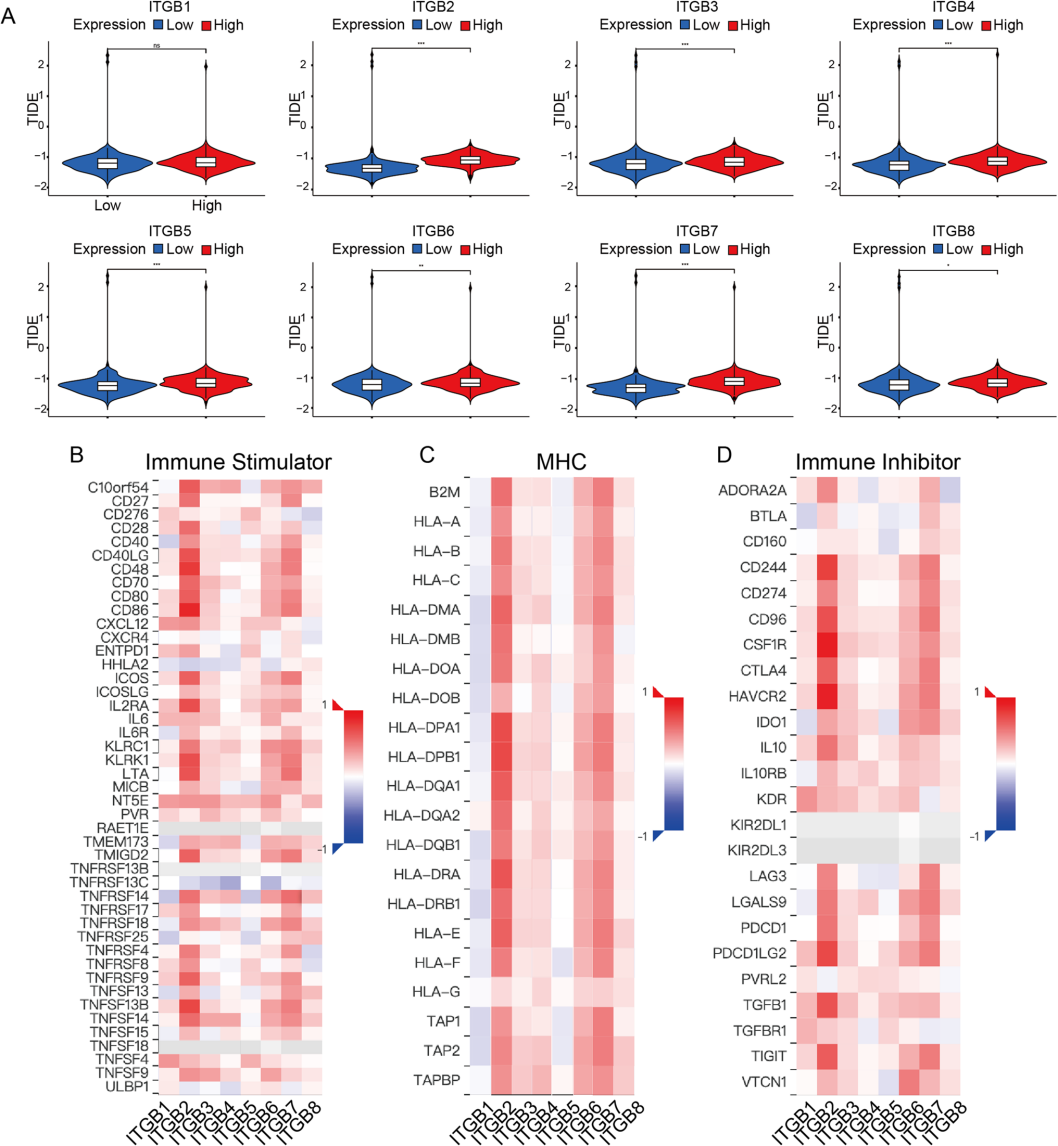
TIDE and immune regulatory analyses of ITGBs. **A** TIDE scores between high- and low-ITGBs groups. High-ITGBs groups had higher TIDE scores than low-ITGBs groups, indicating a high potential to evade anti-tumor immune responses. **B** Correlations between immune stimulators and eight ITGBs which was downloaded from the GSCA database. **C** Correlations between MHC and ITGBs. **D** Correlations between immune inhibitors and ITGBs. MHC, Major Histocompatibility Complex. *, P-value < 0.05; **, P-value < 0.01; ***, P-value < 0.001; ****, P-value < 0.0001

Tumor Immune Dysfunction and Exclusion (TIDE) analysis showed that high expression levels of eight ITGBs members had higher TIDE scores than low expression of ITGBs (Fig. 6A). While TIDE scores represent the ability to evade anti-tumor immune responses, indicating that ITGBs had potential roles in predicting responses to immune therapies. According to a study by Vidotto et al., a set of 63 immune regulators containing immune stimulators, inhibitors, and MHC molecules was used to analyze the correlations with ITGBs in OC (Fig. 6B–D) [36]. Among which, ITGB2, ITGB6, and ITGB7 were positively correlated with immune stimulator-, MHC-, and immune inhibitor-related gene sets, suggesting the potential role of ITGB2, ITGB6, and ITGB7 in influencing immune functions in OC. Moreover, the TIMER database showed that ITGB2 was mostly relevant with tumor-infiltrating immune cells with the |Rho (Spearman's coefficient) ∣ more than 0.5 (Fig.S3). The purity of tumor cells decreased upon the increase of ITGB2, indicating increased immune-cell infiltration with elevated expression levels of ITGB2. According to the immune-cell infiltration analysis, CD4 + T cells, CD8 + T cells, T cell regulatory (Treg) cells, B cells, NK cells, and macrophages were closely positively related to ITGB2. However, significant correlations between ITGB3 and T cell CD4 + , ITGB5 and T cell CD8 + , etc. are driven by outliers (Fig.S3). Therefore, the correlation analyses are only for reference.

### Classification of OC by ITGBs and construction of a predictive model

**Fig. 7:**
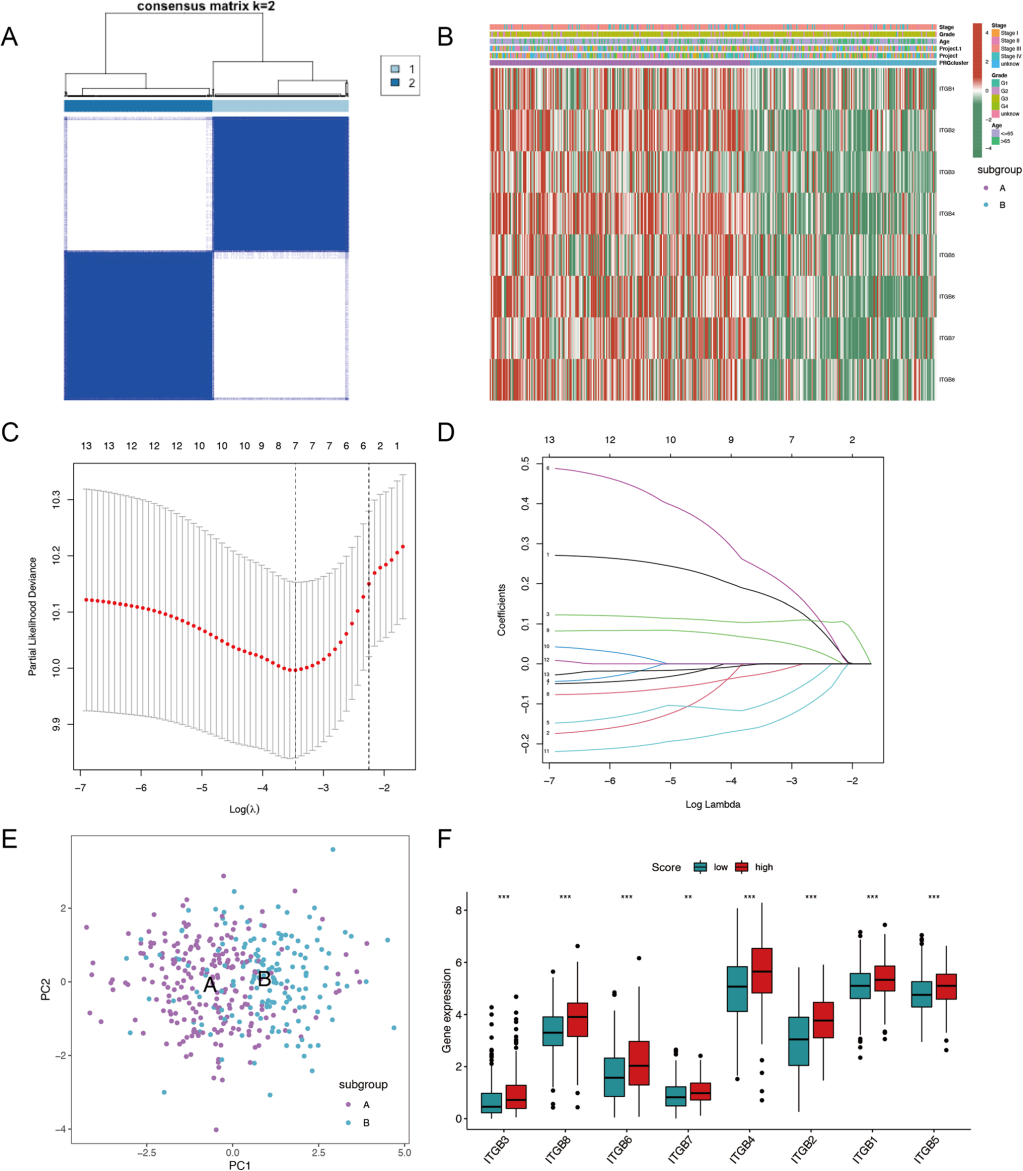
Consensus cluster of ITGBs in OC and predictive model construction. **A** Consensus cluster analysis was performed using the expression matrix of eight ITGBs in the TCGA-OV database. When k = 2, the expression matrix of ITGBs separated the TCGA-OV database into two distinct subgroups, A and B. **B** The expression patterns of eight ITGBs between two subgroups with clinical features in the heatmap. Eight ITGBs decreased in subgroup B. **C** A total of 144 different-expressed genes (FC > 1, P-value < 0.05) between two subgroups were used to construct the predictive model using LASSO regression analysis by the ‘glmnet’ R package. The partial likelihood deviance curve was plotted. The right dotted vertical lines were drawn at the optimal value by using the minimum criteria. **D** According to the optimal value of log (λ), the coefficient of each gene was plotted. **E** PCA distinguished subgroup A from B using the predictive model. **F** Each sample was calculated and scored by the predictive model. Therefore, the predictive model divided the TCGA-OV database into high- and low-score groups. Expression levels of eight ITGBs between high-score and low-score groups which were calculated by the predictive model. *, P-value < 0.05; **, P-value < 0.01; ***, P-value < 0.001

Concerning the above analyses, eight ITGBs were proved to be upregulated in the OC cell line and correlated with poor prognosis of patients with OC. To further investigate the role of ITGBs in OC, subgroup analysis based on the expression matrix of eight ITGBs in the TCGA-OV database was performed using an unsupervised clustering method. It identified two distinct subgroups of the TCGA-OV database, subgroup A and B, (Fig. 7A). Eight ITGBs showed a lower-expression pattern in subgroup B compared with subgroup A (Fig. 7B). Then 144 significantly different-expressed genes (FC > 1, P-value < 0.05) were screened between subgroup A and B. As there are only eight members in the ITGBs family, it was not sufficient for the LASSO regression analysis. Therefore, to construct the predictive model for predicting the prognosis of OC patients, the 114 different-expressed genes were used to construct the predictive model using univariate regression analysis, followed by LASSO regression and multivariate regression analysis (Fig. 7C, D). Before the LASSO regression analysis, samples from the TCGA-OV database were randomly divided into train and test groups. The train group was used to construct the predictive model. The test group was used to verify the efficiency of the predictive model. In the process of constructing the prognostic model, univariate regression analysis of the 114 different-expressed genes was conducted first. Then 13 genes were identified to be associated with the overall survival rate of OC patients and were considered as potential predictive factors for OC patients. Consequently, the LASSO regression analysis and multivariate regression analysis screened 4 genes (AHNAK2, VSIG4, RARRES1, CXCL9) as independent predictive factors to construct the predictive model. The coefficients of the 4 genes were used to calculate the predictive scores of OC samples based on the expression levels of the 4 genes (Table S4) (Predictive score = 0.26265843 * AHNAK2 + 0.32719386 * VSIG4 + 0.10861357 * RARRES1 + (-0.2792445) * CXCL9. OC patients were then divided into high-score and low-score groups according to the median score from the TCGA-OV database. Patients in the high-score group had a poor prognosis and exhibited a high expression pattern of the eight ITGBs. The results showed that the expression levels of the 4 genes can effectively distinguish patients with favorable prognosis from poor prognosis. Despite all eight ITGBs being upregulated in the high-score group associated with poor prognosis, they are not considered as independent prognostic factors for OC patients. Principal component analysis (PCA) displayed the ability of the predictive model to distinguish subgroup A and B (Fig. 7E). The predictive model calculated the predictive score of each sample from the TCGA-OV database. Therefore, the TCGA-OV database was separated into high- and low-score groups. According to the predictive model, eight ITGBs showed an elevated expressional pattern in the high-score group (Fig. 7F).

### Validation of the predictive model which was constructed based on differently expressed genes with ITGBs

**Fig. 8:**
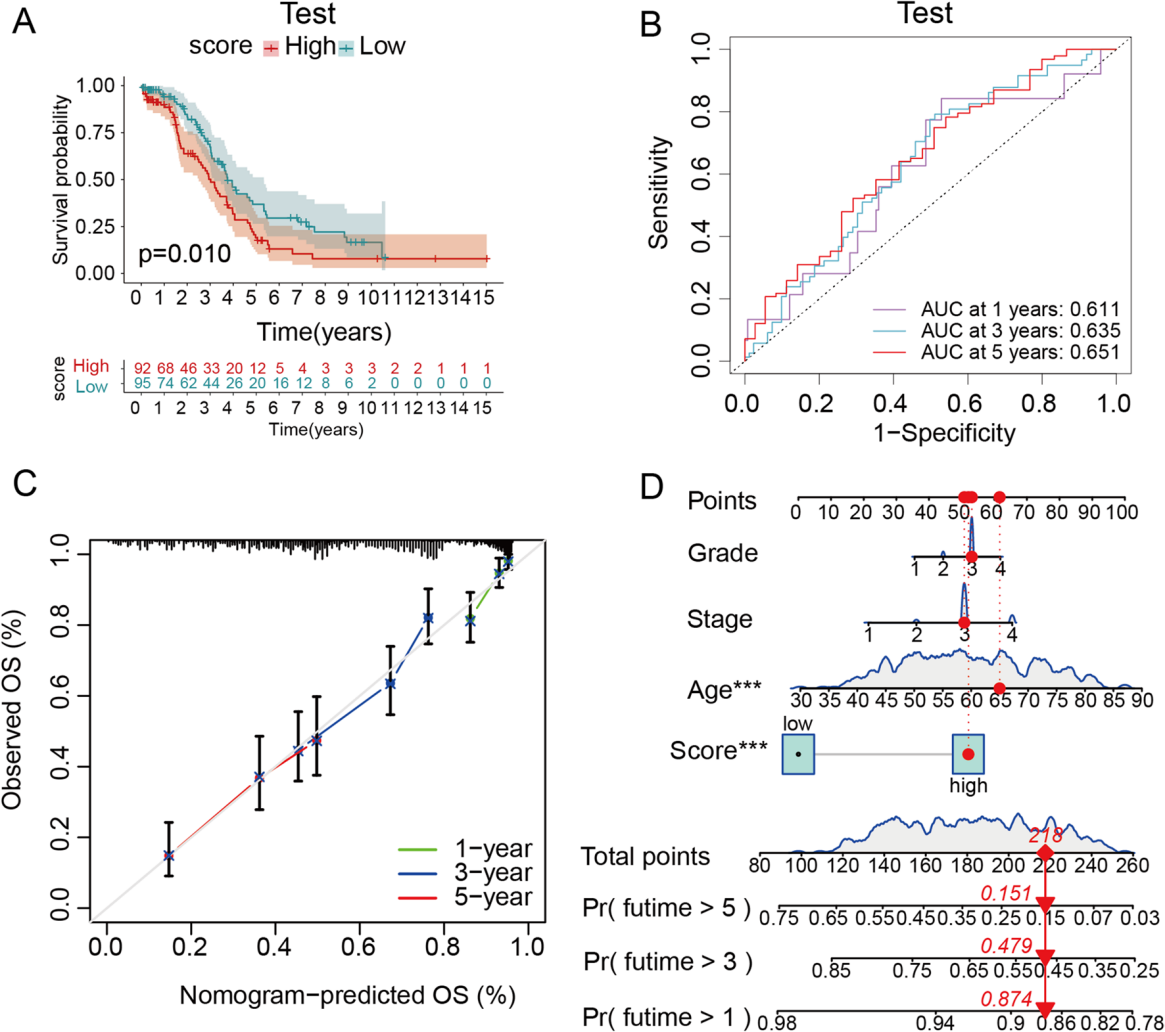
Validation of the predictive model in the test group and nomogram. **A** K-M plot between high- and low-score groups in the test group. **B** The Receiver Operating Characteristics (ROC) curve of the predictive model in the test group showed that the Area Under the Curve (AUC) at 1-, 3-, and 5-year are 0.611, 0.635, and 0.651 respectively. **C** Calibration for nomogram shows the consistency of the 1-, 3-, and 5-year overall survival (OS) rates which were predicted by the predictive model. **D** The nomogram shows an example of how the points were calculated in a representative patient. The survival time over 1-, 3-, and 5-year was predicted using the age, grade, stage, and predictive scores of the TCGA-OV database. The red dots represent points of an OC patient with grade III, stage III, 65-year-old, and high-score status. The total point of the above factors is 218, and the probability of over 5-, 3-, and 1-year survival is 0.151, 0.479, and 0.874, respectively, for this patient. P-value < 0.05 was considered as significant

To verify the efficiency of the predictive model, Kaplan–Meier (K-M) survival analysis, receiver operator curve (ROC) analysis, and nomogram were performed in the test group. Consequent K-M survival analysis showed that the low-score group had a more favorable prognosis than the high-score group in the test group, indicating that the predictive model was able to predict the prognosis of patients with OC (Fig. 8A). Additionally, the ROC analysis of the predictive model demonstrated that it was reliable for predicting the prognosis of patients with OC. The area under the curve (AUC) of 1-, 3-, and 5-year OS rate was 0.611, 0.635, and 0.651 respectively in the test group (Fig. 8B), representing the predictive model had a general performance in predicting the prognosis in the test group. However, the calibration curve of the nomogram analysis showed that the predicted 1-, 3-, and 5-year OS rates were precise according to the standard line (Fig. 8C). Using clinical features, including age, grade, and stage, combined with the predictive model, the nomogram provides a convenient tool for calculating the 1-, 3-, and 5-years OS rate of patients with OC (Fig. 8D).

### Identification and biological functions enrichment of hub genes of ITGBs in OC

**Fig. 9:**
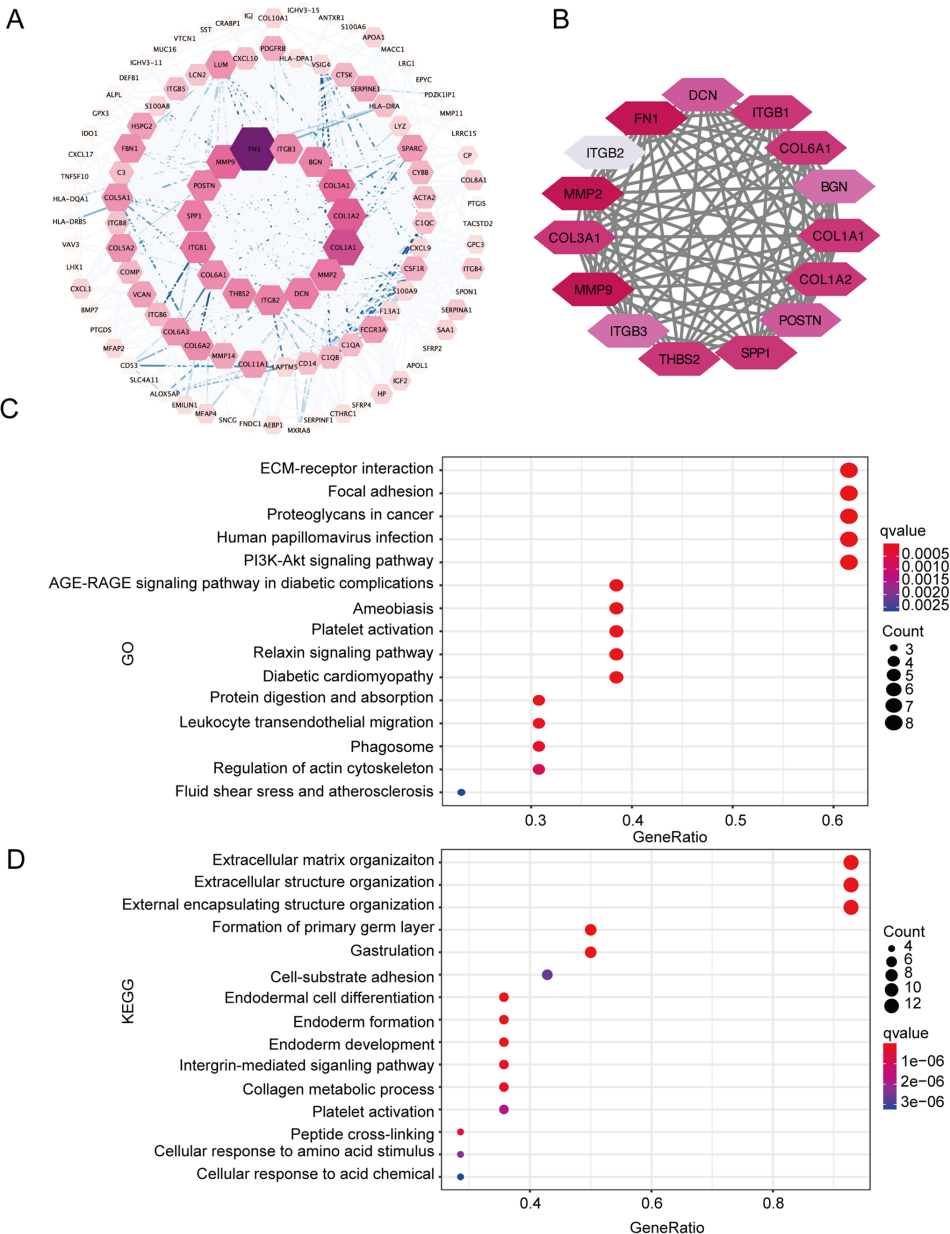
PPI network and hub genes analysis. **A** A total of 268 co-expression genes of eight ITGBs were uploaded onto the STRING database to get the PPI network of eight ITGBs. The PPI network was visualized by the Cytoscape software. The color and size of each gene represent the degree of the corresponding gene. The minimum degree is 1 and the maximum degree is 67. Colored edges represent the co-expression values between genes. Only edges with co-expression value > 0.5 were colored with continuously deepened blue. **B** The top 15 hub genes were identified by degree parameter in the Cytoscape software. **C** GO analysis of the top 15 hub genes. **D** KEGG analysis of the top 15 hub genes

Concerning the above consensus clustering and predictive model based on ITGBs and ITGBs-related genes, it’s interesting to figure out the regulatory relationships of ITGBs in OC. Therefore, a total of 268 co-expression genes of eight ITGBs were uploaded onto the STRING database for a protein–protein interaction (PPI) network. Consequently, the PPI network was plotted by Cytoscape software. The network included 109 nodes and 854 edges (Fig. 9A). To identify the key genes in the PPI network, the degree parameter was calculated using the cytoHubba plugin of Cytoscape software. The top 15 hub genes were identified including FN1, ITGB2, MMP2, COL3A1, MMP9, ITGB3, THBS2, SPP1, POSTN, COL1A1, COL1A2, BGN, COL6A1, ITGB1, and DCN (Fig. 9B), which had more significant roles in the PPI network. Consequently, the top 15 hub genes were uploaded into the DAVID database for GO and KEGG enrichment. GO analysis demonstrated that the top 15 hub genes indeed associated with multiple biological functions, including focal adhesion, PI3K-Akt signaling pathway, and regulation of actin cytoskeleton (Fig. 9C). KEGG analysis showed that extracellular matrix organization and integrin-mediated signaling pathways were enriched, indicating the potential functions of hub genes in metastasis (Fig. 9D). Among the top 15 hub genes, ITGB1, ITGB2, and ITGB3 were ITGB superfamily members. Combined with Univariate Cox analysis, ITGB1 and ITGB3 are promising factors for predicting the prognosis of OC, as well as potential candidates for performing biological functions and regulatory effects in tumorigenesis of OC.

### Prediction of chemo-therapeutic responses of ITGBs in OC

**Fig. 10:**
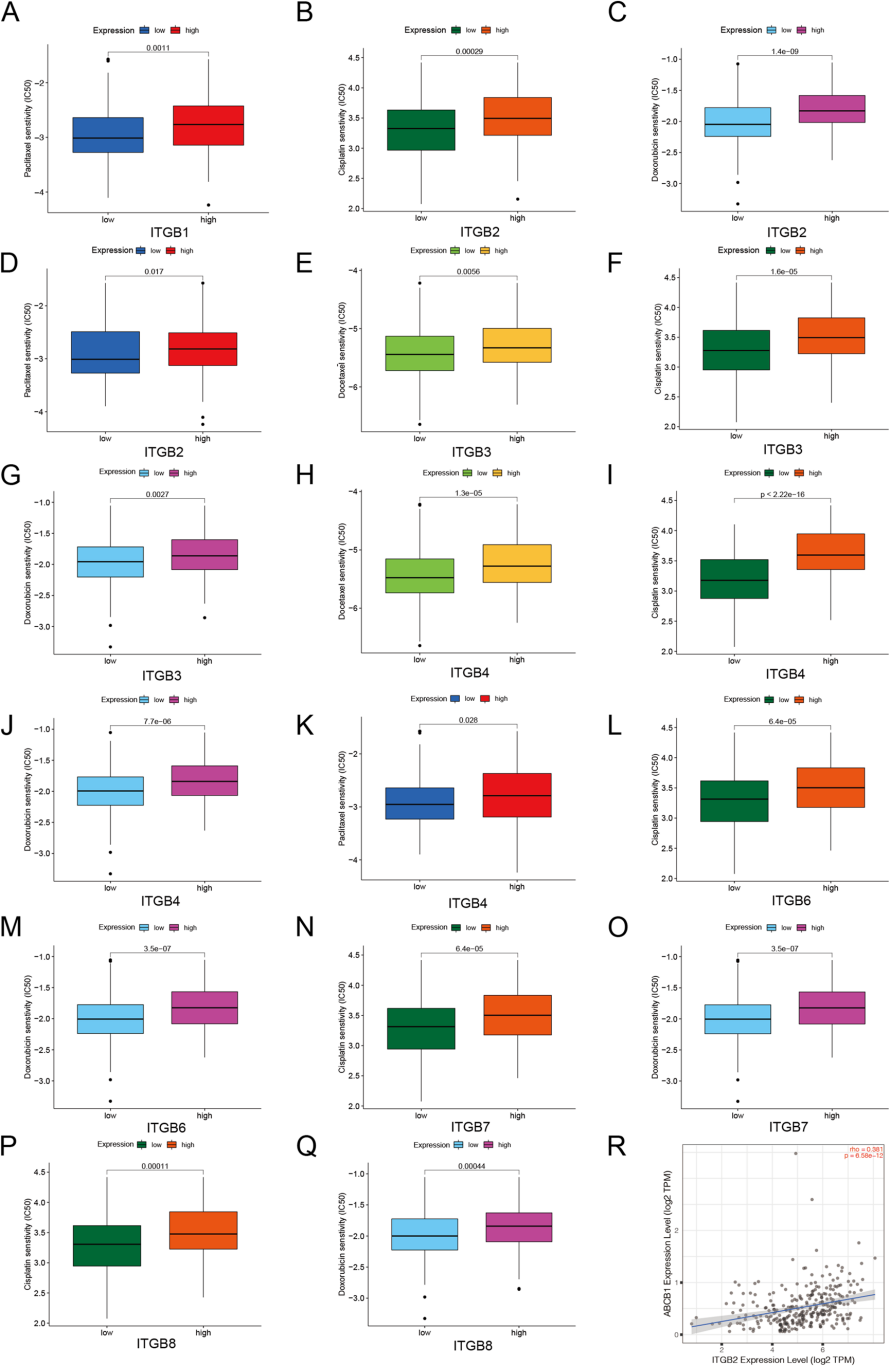
Chemo-therapeutic drug responses of ITGBs. **A**–**Q** Box plot of the IC_50_ of paclitaxel, doxorubicin, docetaxel, and cisplatin between high- and low-ITGB1, ITGB2, ITGB3, ITGB4, ITGB6, ITGB7, and ITGB8 groups. The correlations between ITGBs and IC_50_ chemo-therapeutic drugs were analyzed using the ‘pRRophetic’ R package. **R** Spearman correlation between ABCB1 and ITGB2 was downloaded from the TIMER database. P-value < 0.05 was considered as significant

Additionally, chemo-therapeutic responses of eight ITGBs were predicted using the ‘pRRophetic’ R package. High expression levels of ITGB1 were correlated with paclitaxel resistance (Fig. 10A), high expression levels of ITGB2 with cisplatin, doxorubicin, and paclitaxel resistance (Fig. 10B–D), high ITGB3 (Fig. 10E–G) with docetaxel, cisplatin, and doxorubicin resistance, high ITGB4 with docetaxel, cisplatin, doxorubicin, and paclitaxel resistance (Fig. 10H–K), and high ITGB6, ITGB7, and ITGB8 with cisplatin and doxorubicin resistance (Fig. 10L–Q). ABCB1 is known as a biomarker of multi-chemoresistance, and correlation analysis demonstrated that medium relevance exists between ITGB2 and ABCB1 (Rho > 0.3) (Fig. 10R).

## Discussion

This study provides a general analysis of the prognostic value, biological functions, immune functions, and chemo-therapeutic responses of ITGBs in OC, which is favorable for further investigation of ITGBs in OC. All eight ITGBs were proved to be upregulated in OC cells, whereas ITGB1, ITGB5, ITGB7, and ITGB8 decreased in omental metastasis. IHC images from the HPA database showed increased protein expression levels of ITGB1, 4, 5, 6, and 8 in OC tissues. Subcellular distributions of ITGB1, 2, 5, 7, and 8 are mainly in the cytoplasm. ITGB3 and ITGB4 exist both in the nucleus and cytoplasm, slightly more in the nucleus. However, ITGB6 distributes mainly in the nucleus. The K-M plot showed that higher ITGB1, ITGB3, ITGB4, ITGB5, ITGB6, ITGB7, and ITGB8 levels were correlated with poor prognosis in OC. However, the Univariate Cox analysis showed that only ITGB1, ITGB3, and ITGB8 were risk factors for OC. Currently, studies have verified the prognostic value of ITGB1, ITGB2, ITGB3, and ITGB8 in OC using external databases. Therefore, real-world validation is required [8, 37].

Biological functional enrichment revealed the potential correlation of ITGB1, ITGB3, and ITGB8 with ECM, focal adhesion, and apoptosis. Correspondingly, the knockdown of ITGB1 increased the apoptotic rate and overexpression of ITGB1 led to radio-resistance in non-small cell lung cancer [38–41]. Consistently, ITGB1 has been reported to be involved in lymph node, tumor, and peritoneal metastasis of OC. Moreover, ITGB1 is involved in TGF-β signaling pathway-mediated metastatic behavior of breast cancer and can switch the tumor suppression function of TGF-β to oncogenesis in prostate cancer [42, 43]. As to ITGB3, it was reported to promote metastasis of colorectal cancer, nasopharyngeal carcinoma, pancreatic cancer, and gastric cancer [44]. In contrast, in tumor-repopulating melanoma cells, ITGB3 confers resistance to interferon-α-induced apoptosis by suppressing retinoic acid-inducible gene-I (RIG-I), indicating the involvement of ITGB3 in the anti-apoptotic process [45, 46]. Similarly, ITGB8 has been reported to promote metastasis in colorectal and non-small cell lung cancers [47, 48]. However, the metastatic effects of ITGB3 and ITG8 in OC require further research.

Biological function enrichment of co-expressed genes of eight ITGBs demonstrated that immune responses are correlated with ITGBs as well. ITGB2, ITGB6, and ITGB7 showed distinct correlations with immune regulators including stimulators, inhibitors, and major histocompatibility complex (MHC). All eight ITGBs tended to evade anti-tumor immune responses in OC with higher TIDE scores. As to the predicted immune functions of ITGB1, it has been proven to promote immune escape in pancreatic cancer under the regulation of METTL3-mediated N6-methyladenosine (m^6^A) modification [49]. According to the results of TIMER database analysis, ITGB2 positively correlated with a larger proportion of immune cells including CD4 + T cells, CD8 + T cells, T cell regulatory (Treg), B cells, NK cells, and macrophages. However, further experimental evidence is needed to prove the potential immune regulatory effects of ITGB2 in cancers. Apart from its immune functions, ITGB2 promotes the metastasis of uveal melanoma by regulating ECM signature [50]. The DEL-1/ITGB3 axis is involved in Treg responses during inflammation resolution [51]. Based on the function of ITGB4 in cancer stem cells, immunologic strategies targeting ITGB4 combined with dendritic cells or anti-ITGB4 antibodies armed with tumor-draining lymph node T cells showed benefits in suppressing tumor growth and metastasis in xenograft mouse model [52]. Interestingly, ITGB7 was verified to suppress colorectal cancer pathogenesis by maintaining anti-tumor immunity, whereas it was predicted to evade anti-tumor immunity in OC which requires experimental evidence [52]. Because of the promising efficiency of immune checkpoint inhibitors in advanced OC, it is inspiring to explore the immune functions of ITGB2, ITGB6, and ITGB7 as predicted [6].

To comprehensively understand the biological functions of ITGBs, consensus clustering using the expression matrix of eight ITGBs was used to subgroup the TCGA-OV database. Samples from the TCGA-OV database were successfully divided into distinct two subgroups. Consequently, different-expressed genes between the two subgroups were used to construct a predictive model for the prognostic evaluation of patients with OC. The predictive model divided the TCGA-OV database into high- and low-score groups. Eight ITGBs all increased in the high-score group with an unfavorable OS in OC, indicating biomarker functions of ITGBs in predicting the prognosis of patients with OC. Currently, studies about various predictive models based on different signatures of ovarian cancer provide hints for clinical prognostic evaluation of OC. The signatures conclude gene sets include copper metabolism, immune, pyroptosis, and lncRNA-related gene sets [53–56].

The PPI network revealed 15 hub genes: FN1, ITGB2, MMP2, COL3A1, MMP9, ITGB3, THBS2, SPP1, POSTN, COL1A1, COL1A2, BGN, COL6A1, ITGB1, and DCN. Among these genes, ITGB1, ITGB2, and ITGB3 of ITGB family members are considered to have critical regulatory effects on ITGBs. FN1 is involved in the EMT and metastasis of OC and confers platinum-resistant ovarian cancer-associated mesothelial cells [57, 58]. As to MMP2, the research of MMP2 in OC has been shown to promote metastasis under the regulation of lncRNA TP73-AS1, Nectin-3, β − HCG, and MKL1 [59–62]. To date, COL3A1 has been shown to correlate with poor prognosis in four major types of epithelial ovarian carcinoma patients (high-grade serous, low-grade serous, endometrioid, and mucinous) and was considered a prognostic biomarker for early-stage OC based on a 206 early -stage primary invasive ovarian carcinoma dataset [63]. MMP9 is widely considered as a marker of metastasis in OC [64, 65]. Interestingly, a study showed that SPP1 promoted OC progression via the ITGB1/FAK/AKT pathway and was identified as a biomarker for OC prognosis and progression of OC [66, 67]. Additionally, SPP1-positive T cells represent malignant progression, poor prognosis, and suppression of immune checkpoints in OC [68]. Consistent with the PPI network, POSTN-ITGB1 promoted metastasis of OC under the regulation of DDR2 and TGF-beta as well [39, 69]. Moreover, POSTN is involved in stemness and recurrence of OC [70]. COL1A1 has been reported to be involved in the progression, metastasis, and carboplatin resistance of OC [71–73]. Biological function enrichment based on interactive genes with ITGBs revealed the potential role of ITGBs in metastasis, the PI3K-Akt signaling pathway, phagosome, and regulation of the actin cytoskeleton. ITGB2 was identified as a prognostic marker of OC [37]. However, ITGB2 was not found to be significantly correlated with the prognosis of OC in this study. ITGB4 is related to migration and invasion in OC cell lines, which was further validated in vivo [74]. Consistent with our prediction, this study demonstrated that ITGB6 promotes metastasis and resistance to cisplatin in OC both in vivo and in vitro [75]. Similarly, another study showed that the ITGB6/TGF-beta axis performed a promotion effect on the invasion and adhesion of OC spheroids [76]. ITGB8-mediated cisplatin resistance was counteracted by miR-199a-3p in OC [77]. Currently, no experimental evidence has demonstrated the biological functions of ITGB2, ITGB3, and ITGB5, and no studies have focused on ITGB7.

Regarding common chemo-therapeutic drugs, paclitaxel, cisplatin, docetaxel, and doxorubicin, high ITGB1 tended to resist paclitaxel treatment, high ITGB2 tended to resist paclitaxel, cisplatin, and docetaxel treatment, ITGB3 to cisplatin, docetaxel, and doxorubicin, ITGB4 to paclitaxel, cisplatin, docetaxel, and doxorubicin, and ITGB7 and ITGB8 to cisplatin and doxorubicin. ITGB1 was verified to promote various chemo-therapeutic resistance in cancer types, including tamoxifen resistance and epirubicin resistance in breast cancer cells, docetaxel resistance in esophageal squamous cell carcinoma, bevacizumab resistance in glioblastoma, osimertinib resistance inhibitor resistance in non-small cell lung cancer, and paclitaxel resistance in nasopharyngeal cancer [78–84]. Moreover, ITGB1 regulates the radio-resistance of oral squamous carcinoma cells as well [85]. However, the role of ITGB1 in the chemoresistance of OC requires further investigation. ITGB3 promotes cisplatin resistance in osteosarcoma [86] and is abundant in both drug resistance and the mesenchymal status of mesenchymal lung cancer [87]. In the regulatory effect of lipocalin 2, ITGB3 conferred resistance to 5-fluorouracil in colorectal cancer [88]. Exosome-transmitted FOSL1 is attributed to oxaliplatin resistance in colorectal cancer via activation of ITGB4 [89]. As previously described, ITGB4 is present on the surface of cancer stem cells. Therefore, immunotherapy combined with ITGB4-targeted therapy showed a satisfactory effect [52]. ITGB8 has been shown to confer paclitaxel and cisplatin resistance in OC and paclitaxel resistance in lung cancer [77, 90, 91]. In addition, it is related to Lenvatinib and gefitinib resistance in hepatocellular carcinoma [92, 93].

To our knowledge, this is the first study to analyze the biological functions and chemo-therapeutic responses of ITGBs in OC patients. All eight ITGBs were upregulated in OC, and ITGB1, ITGB3, and ITGB8 were risk factors for OC. Combined with biological analysis, it was promising to investigate the metastatic function of ITGBs in OC. Immune therapy is emerging to benefit patients with OC. Since ITGB2 is strongly correlated with immune cells, it is possible to investigate the role of ITGB2 in the tumor microenvironment and immune cell infiltration of OC. The most relevant factor influencing the prognosis of patients with OC is chemoresistance. Therefore, the role of ITGB1, ITGB2, ITGB6, and ITGB7 in paclitaxel and cisplatin resistance in OC needs to be elucidated.

## Conclusion

This paper describes the expression levels, subcellular distribution, prognostic value, predictive model, biological functions, regulatory effects, immune functions, and chemo-therapeutic response of eight ITGBs in OC. It provides promising ideas for diagnosis, prediction of prognosis, monitoring of therapeutic responses, and potential therapeutic targets of ITGBs in OC.

### Supplementary Information

Supplementary Material 1 (DOCX 17728 KB) Table S1. Information of the GEO Datasets involved in this study. Table S2. Sequences of primers for qRT-PCR. Table S3. Univariate regression analysis screened 13 genes associated with the overall survival rate of OC patients. Table S4. Coefficients of the 4 genes of the predictive model. Figure S1. Workflow of the study. Figure S2. Batch effect removing of GSE131978 from GPL570 and GPL96. Figure S3. TIMER of ITGBs in OC.

## Data Availability

The data that support the findings of this study are available in the TCGA database at https://portal.gdc.cancer.gov/ and GEO DataSets at https://www.ncbi.nlm.nih.gov/gds/, reference number [8]. These data were derived from the following resources available in the public domain: https://www.ncbi.nlm.nih.gov/geo/query/acc.cgi?acc=GSE26712; https://www.ncbi.nlm.nih.gov/geo/query/acc.cgi?acc=GSE133859; https://www.ncbi.nlm.nih.gov/geo/query/acc.cgi?acc=GSE131978.
