# Supplementary material for: Identification of the biological functions and chemo-therapeutic responses of ITGB superfamily in ovarian cancer
Source: Discov Oncol. 2024 May 30;15:198. doi: 10.1007/s12672-024-01047-4 (PMC11139846; doi:10.1007/s12672-024-01047-4)
Supplement: Supplementary file 1 — Supplementary Material 1 (DOCX 17728 KB) Table S1. Information of the GEO Datasets involved in this study. Table S2. Sequences of primers for qRT-PCR. Table S3. Univariate regression analysis screened 13 genes associated with the overall survival rate of OC patients. Table S4. Coefficients of the 4 genes of the predictive model. Figure S1. Workflow of the study. Figure S2. Batch effect removing of GSE131978 from GPL570 and GPL96. Figure S3. TIMER of ITGBs in OC. [file 12672_2024_1047_MOESM1_ESM.docx]

**Supplementary Table 1**. Information of the GEO DataSets involved in this study

| GEO series | Contributors, Year | Samples (n) | GEO Platform # |
| --- | --- | --- | --- |
| GSE26712 | Birrer MJ, 2011 | 195 | GPL96 |
| GSE133859 | Song F, etc, 2020 | 28 | GPL16699 |
| GSE131978 | Ravaggi A, etc, 2019 | 39 | GPL96 and GPL570 |

EOC, epithelial ovarian cancer; GEO, Gene Expression Omnibus; GPL, GEO Platform; n, number of samples.

**Supplementary Table** 2. Sequences of primers for qRT-PCR

| Genes | Sequences |
| --- | --- |
| ITGB1-F | GTAACCAACCGTAGCAAAGGA |
| ITGB1-R | TCCCCTGATCTTAATCGCAAAAC |
| ITGB2-F | TGCGTCCTCTCTCAGGAGTG |
| ITGB2-R | GGTCCATGATGTCGTCAGCC |
| ITGB3-F | CATGAAGGATGATCTGTGGAGC |
| ITGB3-R | AATCCGCAGGTTACTGGTGAG |
| ITGB4-F | CTCCACCGAGTCAGCCTTC |
| ITGB4-R | CGGGTAGTCCTGTGTCCTGTA |
| ITGB5-F | AACTCGCGGAGGAGATGAG |
| ITGB5-R | GGTGCCGTGTAGGAGAAAGG |
| ITGB6-F | GAGGACTACCCGGTGGATTTG |
| ITGB6-R | TCCTTTATTGTGTTGAGGTCGTC |
| ITGB7-F | TGGACCTGAGCTACTCCATGA |
| ITGB7-R | GGTGAAAGCTGAATGGTGACTG |
| ITGB8-F | CGTGACTTTCGTCTTGGATTTGG |
| ITGB8-R | TCCTTTCGGGGTGGATGCTAA |
| ACTB-F | TCATCACCATTGGCAATGAG |
| ACTB-R | CACTGTGTTGGCGTACAGGT |

F, forward primer; R, reverse primer.

**Supplementary Table** 3. Univariate regression analysis screened 13 genes associated with the overall survival rate of OC patients

| Genes | HR | HR.95L | HR.95H | P-value |
| --- | --- | --- | --- | --- |
| AHNAK2 | 1.17859787 | 1.04756288 | 1.32602344 | 0.00628199 |
| ALOX5AP | 1.10608546 | 1.00459873 | 1.2178246 | 0.04003329 |
| PTGER2 | 1.19333262 | 1.06481396 | 1.33736296 | 0.00236468 |
| TGFBI | 1.17787532 | 1.0492181 | 1.32230874 | 0.00553573 |
| GBP4 | 0.87774984 | 0.79665992 | 0.96709369 | 0.00837636 |
| VSIG4 | 1.16319952 | 1.04718013 | 1.29207295 | 0.00480389 |
| SLC4A11 | 1.10952068 | 1.0051384 | 1.22474292 | 0.0392426 |
| AC004540.2 | 0.90187103 | 0.82013952 | 0.99174754 | 0.03309458 |
| RARRES1 | 1.08000769 | 1.00817408 | 1.15695954 | 0.02839487 |
| CXCL10 | 0.92243789 | 0.86487697 | 0.98382971 | 0.01405463 |
| CXCL9 | 0.89058651 | 0.82793551 | 0.95797839 | 0.00184911 |
| IGKC | 0.95703916 | 0.92161881 | 0.99382081 | 0.02248347 |
| IGHG1 | 0.95066696 | 0.91244065 | 0.99049475 | 0.01568892 |

HR, hazard ratio. HR.95L, lower 95% confidence interval. HR.95H, upper 95% confidence interval.

**Supplementary Table** 4. Coefficients of the 4 genes of the predictive model

| Genes | Coefficient |
| --- | --- |
| AHNAK2 | 0.26265843 |
| VSIG4 | 0.32719386 |
| RARRES1 | 0.10861357 |
| CXCL9 | -0.2792445 |

Predictive score = 0.26265843 * AHNAK2 + 0.32719386 * VSIG4 + 0.10861357 * RARRES1 + (-0.2792445) * CXCL9.


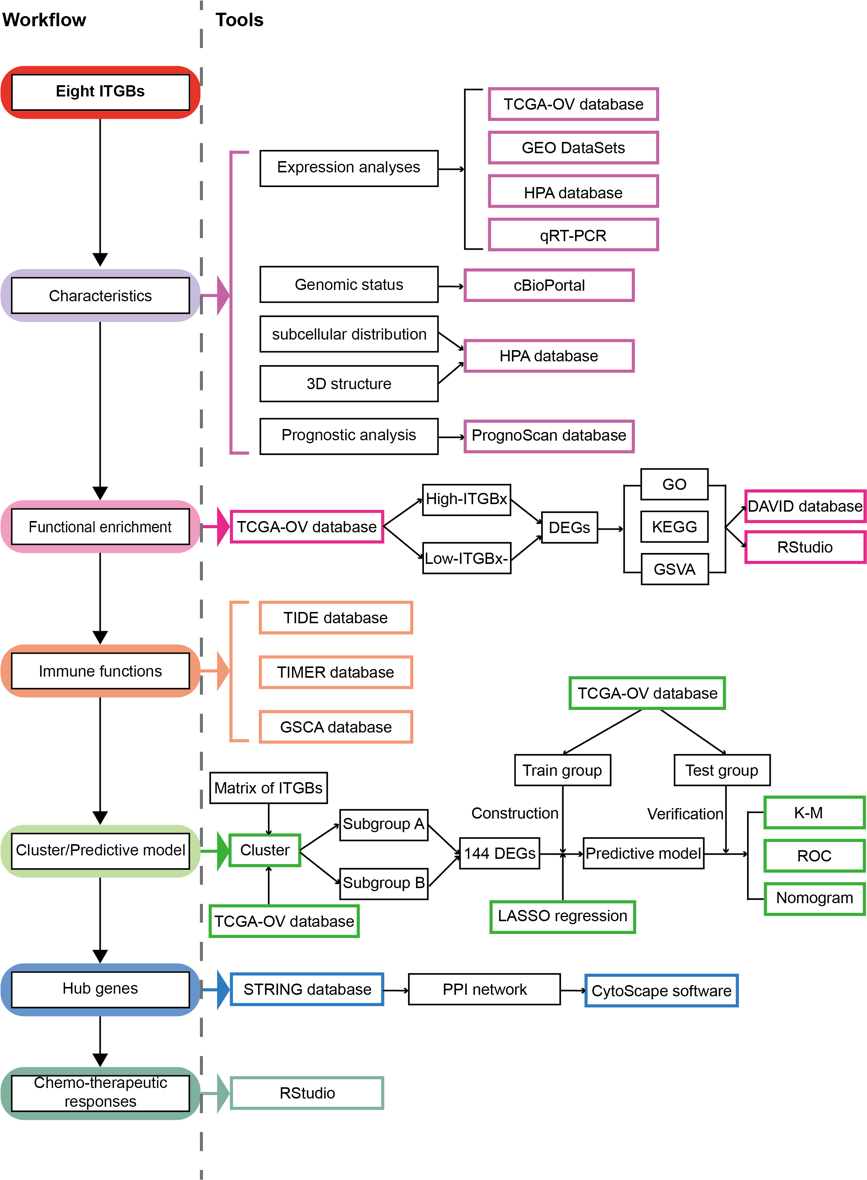


**Supplementary Figure 1**. Workflow of the study. The left flowchart beside the dotted line is the goal of the analysis. The right workflow represents the tools used for the corresponding goal. ‘x’ in High-ITGBx and Low-ITGBx is number 1-8. The TCGA-OV database was divided into High-ITGBx and Low-ITGBx according to the median value of ITGBx in the TCGA-OV database. Text with colored outlines represents tools and databases. DEGs, different-expressed genes. Cluster, Consensus clustering analysis.


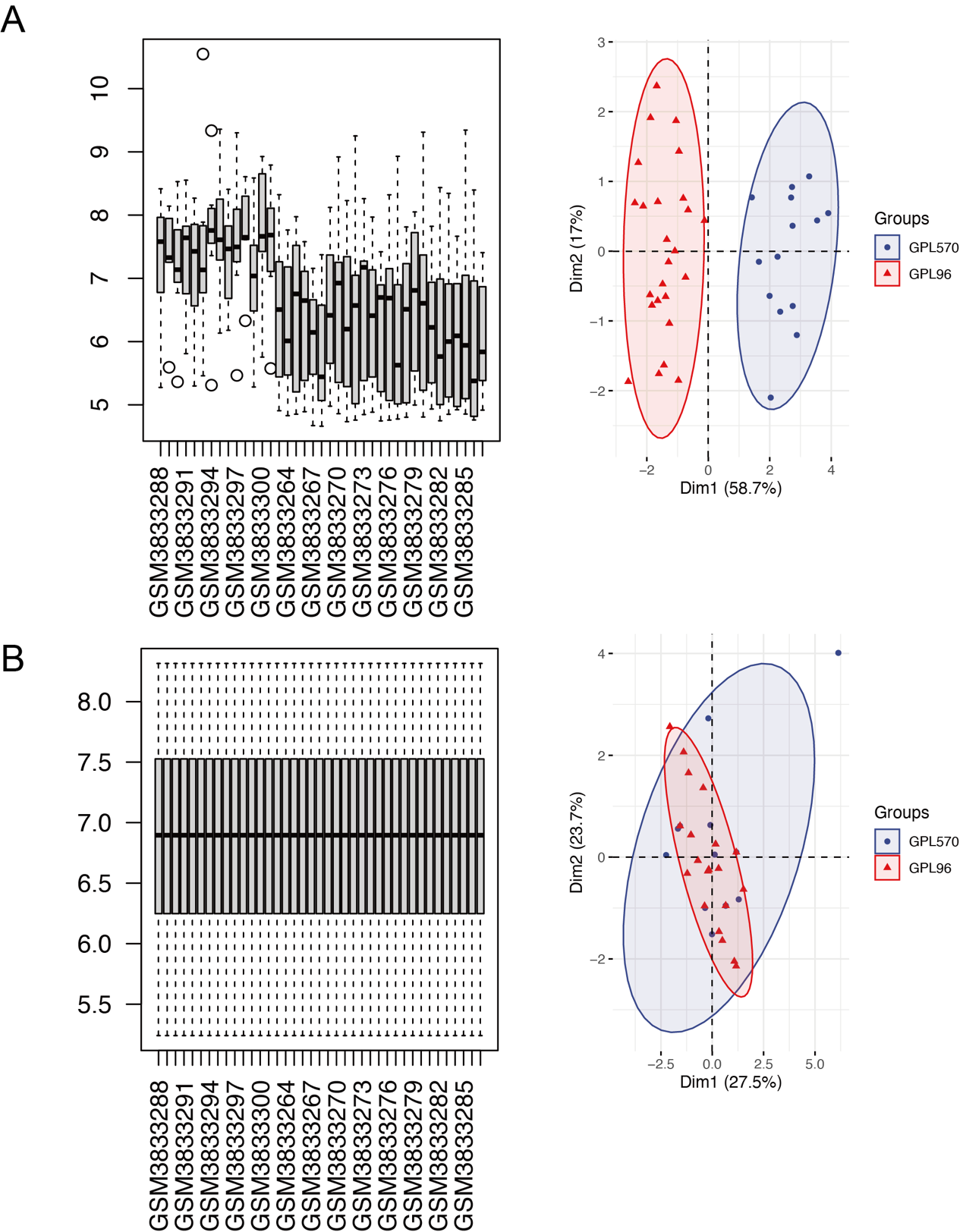


**Supplementary Figure 2**. Batch effect removing of GSE131978 from GPL570 and GPL96. (A) Bar plot and principal component analysis (PCA) of GSE131978 from GPL570 and GPL96 before removing batch effect. (B) After removing the batch effect, the bar and PCA plot showed excellent consistency and the data of GSE131978 was comparable.


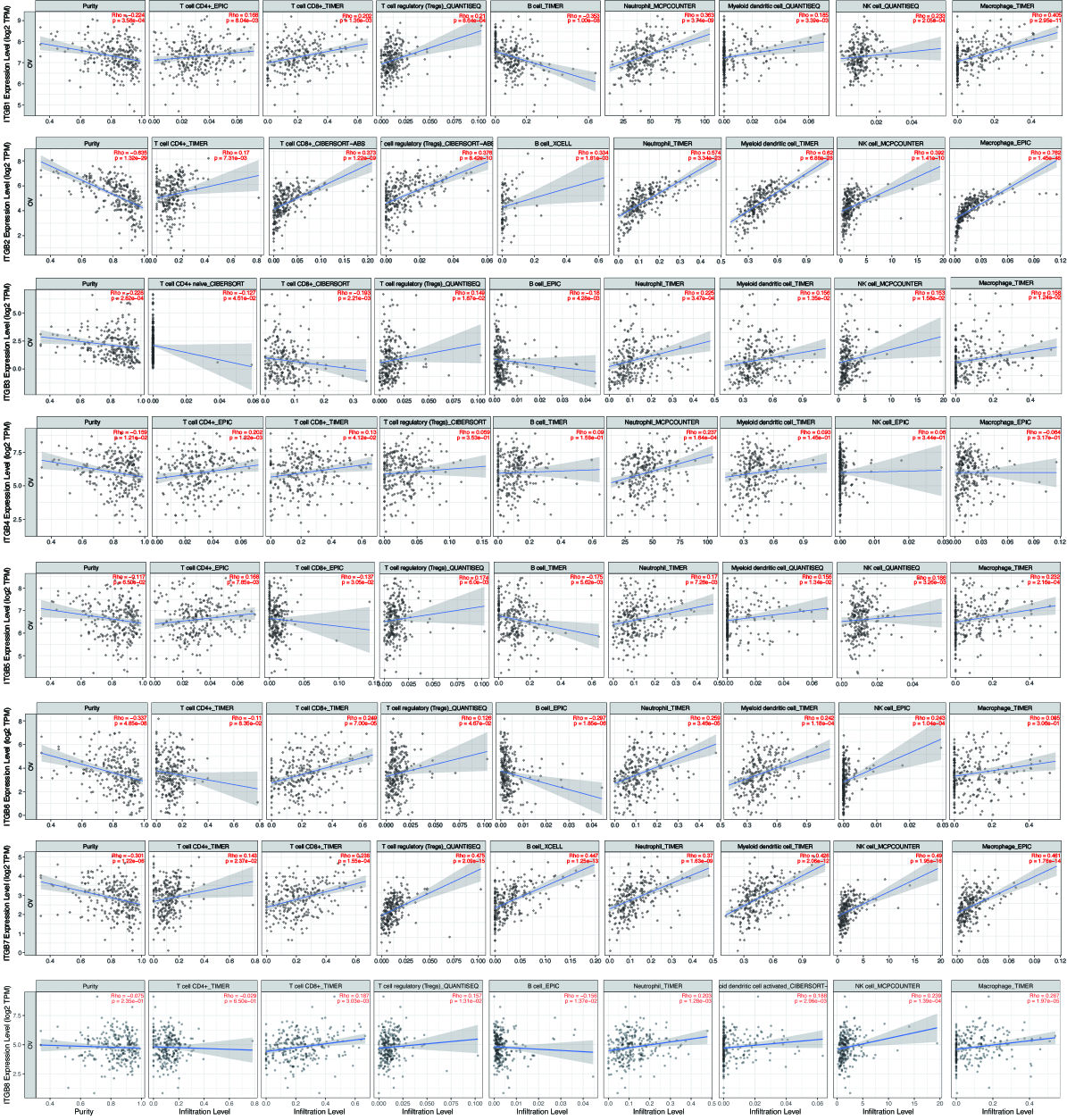


**Supplementary Figure 3**. TIMER of ITGBs in OC. Correlation between tumor cell purity, CD4+ T cell, CD8+ T cell, NK cell, Treg cell, B cell, Neutrophil cell, myeloid dendritic cell, macrophage cell, and eight ITGBs in OC from TIMER database. Some significance is caused by outliers.
